# Exploring the biological behavior and underlying mechanism of KITLG in triple-negative breast cancer

**DOI:** 10.7150/jca.90051

**Published:** 2024-01-01

**Authors:** Liuqing Qin, Yuchao Li, Yifei Huang, Chaoyi Tang, Wenkang Yang, Yujun Tang, Caixin Qiu, Min Mao, Jiehua Li

**Affiliations:** 1 Department of Gastrointestinal Gland Surgery, The First Affiliated Hospital of Guangxi Medical University, Nanning 530021, Guangxi, China.; 2 The Thyroid and Breast Surgery Department, The First People's Hospital of Qinzhou, Qinzhou 535000, Guangxi, China.

**Keywords:** triple-negative breast cancer, KITLG, biological behavior, mechanism

## Abstract

The tyrosine-kinase receptor that is specified by the KIT locus is demarcated by KITLG. This multifaceted factor is instrumental during in-utero germ and neural cell maturation and hematopoiesis, ostensibly reflecting its role in facilitating cell migration. Concurrently, KITLG is prone to a mutation in germ cell tumors, entailing a presumed connection to tumorigenesis. Despite this, the intricacies of its function in breast cancer and the relevant mechanisms remain elusive. Multiple independent databases depict a consistently low expression of KITLG within tissues affected by triple-negative breast cancers (TNBC), a trend strongly coupled with reduced survival rates. Interestingly, non-triple-negative breast cancers exhibit a markedly high expression of KITLG compared to the norm. An initial analysis of the GEO database speculates that KITLG may serve as an oncogene suppressor in TNBC, hinting at varied roles for KITLG isoforms within this disease context. In conclusion, our preliminary analysis offers valuable insights into the role and expression pattern of KITLG in TNBC. We provide evidence supporting its consideration as a promising new prognostic marker, thereby potentially enriching therapeutic strategies for TNBC. Indeed, given the limited advances in molecularly targeted therapy for TNBC, a significant need exists for a more precise therapeutic approach and a comprehensive understanding of its inherent mechanisms of action.

## Introduction

TNBC is a unique immunohistochemical subtype of breast cancer, characterized by minimal expression of estrogen receptor (ER), progesterone receptor (PR), and human epidermal growth factor receptor 2 (HER-2). This malignancy is often associated with aggressive disease progression, high potential for metastasis, increased instances of recurrence, and lamentable prognosis^1^. This means that TNBC does not respond to hormone therapies or targeted therapies that are effective for other types of breast cancer. The lack of these receptors also makes TNBC more aggressive and difficult to treat. The propensity for early local recurrence and distant metastasis act as contributing factors^2^ to treatment failure and subsequent mortality in TNBC cases, leading to death rates as high as 40% within the first five years post-diagnosis^3^. Consequently, the exploration and development of innovative therapeutic strategies for TNBC emerge as a critical clinical imperative.

c-KIT Ligand (KITLG), commonly known as Stem Cell Factor (SCF), is a potent cell growth regulator. Acting primarily as a ligand by binding to c-KIT, it presents significant biological activities on an array of tissue cells^4^-^6^, including hematopoietic stem cells, pigment cells, germ cells, and tumor cells. Evidence suggests that KITLG performs an essential role in numerous cancers. For instance, studies indicate that in nasopharyngeal carcinoma, KITLG potentially orchestrates the proliferation, invasion, and metastasis of cancer cells both in vitro and in vivo, via the JAK/STAT signaling pathway^7^. Similarly, in bladder cancer, KITLG is purported to modulate T24 cells through TLR, NF-κappa B, TNF, or TGF-β signaling pathways, regulated by ERH genes^8^. Additionally, in testicular germ cell tumors^9^, a comprehensive examination of the signaling pathways within their genetic risk factors and pathogenesis underscores that the KITLG/c-KIT pathway is pivotal in its evolution.

Our preliminary research elucidates a strong correlation between KITLG and apoptosis, thereby implicating KITLG as a potential participant in the apoptotic pathway as well. We uncovered that overexpression of KITLG instigated an increase in the expression of multiple genes within the apoptotic pathway, thereby enhancing apoptosis. Furthermore, this overexpression facilitated the transcription of BCL-2/BAX and Casp3/9 but inhibited the expression of AKT/PI3K^10^. This suggests a mechanism where apoptosis acceleration could occur, leading to a setback in the advancement of triple-negative breast cancer cells.

The findings of this investigation have brought attention to the significance of the atypical expression of KITLG as a prospective focus for managing triple-negative breast cancer. This finding suggests that by focusing on regulating KITLG expression, it may be possible to develop a more efficient and effective treatment for this particular type of breast cancer. This discovery offers hope for patients and researchers in the field, as it presents a new avenue for drug development and therapy.

## Materials and methods

### Collection and processing of database information

A total of six expression Breast cancer cohorts came from Gene Expression Omnibus (GEO, http://www.ncbi.nlm.nih.gov/geo/) by using the GEO query R package, including GSE65216, GSE45827, GSE37751, GSE43358, GSE76275, and GSE27447 (Table S1). The ER-, PR-, and HER2- were identified as TNBC in these cohorts. The expression of KITLG in these cohorts was compared to normal breast tissues or non-TNBC tissues using an independent t-test. Survival data of 107 triple-negative breast cancer (GSE103091) were downloaded from the GEO dataset. The impact of KITLG on the survival of triple-negative breast cancer was assessed using the Kaplan-Meier survival curve analysis. In order to conduct this analysis, the samples were divided into two groups: a high KITLG expression group and a low KITLG expression group. The division was made based on the median KITLG expression value. The association of clinical features and KITLG expression level was tested through a t-test or ANOVA test.

### Clinical specimen collection

Forty-three triple-negative breast cancer tissues and matching para-cancer tissues were collected from patients who underwent surgery in the Second Ward of Gastrointestinal Glandular Surgery of the First Affiliated Hospital of Guangxi Medical University between June 2022 and December 2023. The inclusion criteria for the cancer group were as follows: 1) All patients were diagnosed for the first time. Immunohistochemistry and pathologically diagnosed as triple-negative breast cancer, ER-, PR-, HER2-; 2) Patients treated with surgical resection; and 3) Patients who had not received preoperative anti-cancer treatment, such as chemotherapy or radiotherapy. The exclusion criteria were patients with a history of other malignant diseases and chronic system diseases. The specimens were immediately placed in lyophilized tubes with RNA protection solution and transferred to small liquid nitrogen tanks for storage. This study was approved by the "Ethics Committee of Guangxi Medical University".

### Reagents and antibodies

Dul becco's Modified Eagle Medium (DMEM), Fetal Bovine Serum (FBS), Penicillin/Streptomycin antibiotic, CCK-8, Annexin V-FITC/PI apoptosis kit. Primary antibodies for KITLG (Proteintech, 26582-1-AP), β-Actin (Proteintech), P-AKT (CST), PI3K (CST, 4257S), c-KIT, BCL-2 (Affinity, AF6139), BAX (Affinity, AF0120), Caspase-3 (CST, 9662S), Caspase-9 (CST, 9502T), cleaved-Caspase9 (CST, 9505T), Cyt-c, and Goat anti-Rabbit IgG (H+L) Secondary Antibody, DyLight™ 800 4X PEG (Invitrogen, SA535571) were used for blotting.

### Cell culture

MDA-MB-231 cells were obtained from Procell Life Science & Technology Co., Ltd. They were cultivated in DMEM medium enriched with 10% FBS and 1% penicillin/strepto mycin antibiotic. MCF-10A cells were also used in this study. They were cultured in DMEM/F 12 supplemented with 20ng/ml EGF, Hydrocortisone, Insulin, NEAA, 5% HS, and 1% penicillin/streptomycin Solution. The manufacturer's recommended protocols were followed for maintaining the cells. All cell cultures were maintained in a humid incubator at 37°C with 5% CO2.

### Cell transfection and virus infection

To overexpression, KITLG, LV-KITLG (82788-1) (SHANGHAI GENECHEM CO., LTD) was used. Cell lines expressing stable shRNA were acquired by infecting breast cancer cells with lentivirus particles, and subsequent selection with puromycin for a duration of 1 week. Validation of the efficiency of overexpression was conducted through qRT-PCR or Western Blotting.

### CCK-8 assays

The MDA-MB-231 TNBC cell line was placed into 96-well plates (2000 cells per well) and allowed to adhere for 12 hours prior to transfection. Cell proliferation was evaluated using CCK-8 assays (YEASEN, China) at 24-hour intervals starting from day 1 until 72 hours following transfection.

### Cell apoptosis assay

In order to assess the influence of KITLG on the apoptosis of TNBC cells, we employed the Annexin V-APC/7-AAD apoptosis kit (manufactured by Lianke, located in Hangzhou, China). The procedure was executed following the instructions provided by the supplier.

### Wound-healing, transwell migration and invasion assays

For the wound-healing analysis, a total of 200,000 cells were seeded onto a 6-well plate. Once reaching complete confluence, an artificial "wound" was created in the middle of the culture plate using a 100 μl pipette tip specifically for the MDA-MB-231 cells. The rate of wound healing was measured by comparing the distance of the healed wound to the original wound at 0 and 24 hours. Regarding the transwell experiment, transwell chambers with an 8 μm pore size from JET BIOFIL were utilized to assess both cell migration and invasion. The lower chamber was filled with a culture medium supplemented with 10% FBS. The breast cancer cells, 100,000 in total (MDA-MB-231), were suspended in a serum-free medium and then placed in the upper chamber. After 24 hours of incubation, the cancer cells present in the upper chamber were carefully removed using a cotton swab. The cells that successfully penetrated and attached to the bottom filter were fixed using a mixture of 95% ethanol in PBS. Subsequently, the fixed cells were stained with 0.5% crystal violet for 30 minutes before being imaged using a 10× objective lens. The statistical analysis of invasion cell numbers was performed based on data obtained from three separate experiments, with an average taken from five image fields.

### Isolation of RNA and qRT-PCR

RNA was isolated from treated cells using the RNA simple Total RNA Kit (TianGen, China). Reverse transcription was carried out using Prime ScriptTM RT Mix (TaKaRa) according to the recommended protocols provided by the manufacturer. Primers were designed based on the information available on the Primer Bank website. Quantitative real-time polymerase chain reaction (qRT-PCR) was conducted using SYBR Premix Ex Taq (TAKARA) on a 7500 Fast Real-Time PCR System (Applied Biosystems). The mRNA expression levels obtained from qRT-PCR data are presented relative to the expression levels of the reference gene. The internal control used for this purpose was Glyceraldehyde-3phosphate dehydrogenase (GAPDH), and the 2^-△△Ct^ method was applied to measure gene expression. The gene-specific primers utilized can be found in the Supplementary material.

Expressions of designated genes were determined by quantitative real‐time PCR. All reactions were run in triplicate.

### Western blot and Protein extraction

To obtain total cell lysates, the cells were subjected to a thorough wash with ice‐cold PBS, after which they were lysed in RIPA buffer. The RIPA buffer was supplemented with protease and phosphatase inhibitors, and the lysation process lasted for 10 minutes. To eliminate any cell debris, the lysate was centrifuged at 16000 rpm for 10 minutes at 4°C. Subsequently, 20 μg of the whole cell lysate was subjected to electrophoresis using a denaturing 10% SDS‐polyacrylamide gel. The gel was then transferred to a membrane, which was further used for blotting with specific antibodies.

### Animal Studies

A total of fifteen female BALB/c nude mice, aged 3 to 4 weeks, weighing between 18 and 20 grams, were obtained from the Experimental Animal Center of Guangxi Medical University. The mice were housed in animal rooms meeting SPF-grade standards, with an ambient temperature ranging from 20 to 26°C and relative humidity between 40% and 60%. A light/dark cycle of 12 hours each was maintained. MDA-MB-231 cells, transfected with either KITLG overexpression-lentivirus or NC-lentivirus, were injected into the subcutaneous armpit of the right forelimb of each mouse (n=5 per group). Tumor growth was monitored weekly for a period of 1 month following injection, and tumor volume was calculated using the formula: V = length × width^2 × 0.5. All animal experiments were performed in compliance with international animal ethics requirements and were approved by the Medical Ethics Committee of Guangxi Medical University.

### Software and statistical analysis

Data were processed in the bioinformatics section using R open-source statistical software, and data were processed and expressed as Mean ± standard deviation (mean ± SD) was used to express the data. The real-time fluorescence quantitative PCR and WB protein immunoblotting sections were statistically analyzed using the statistical software SPSS22. 0, and the data were expressed as mean ± standard deviation (x ± s). Means between two groups of data were compared using the t-test, and means between multiple samples were compared using one-way ANOVA with post hoc comparisons using the L-LSD method, with P < 0. 05 considered statistically significant.

## Results

### Low expression of KITLG in TNBCs and associated with poor prognosis

**Figure 1:**
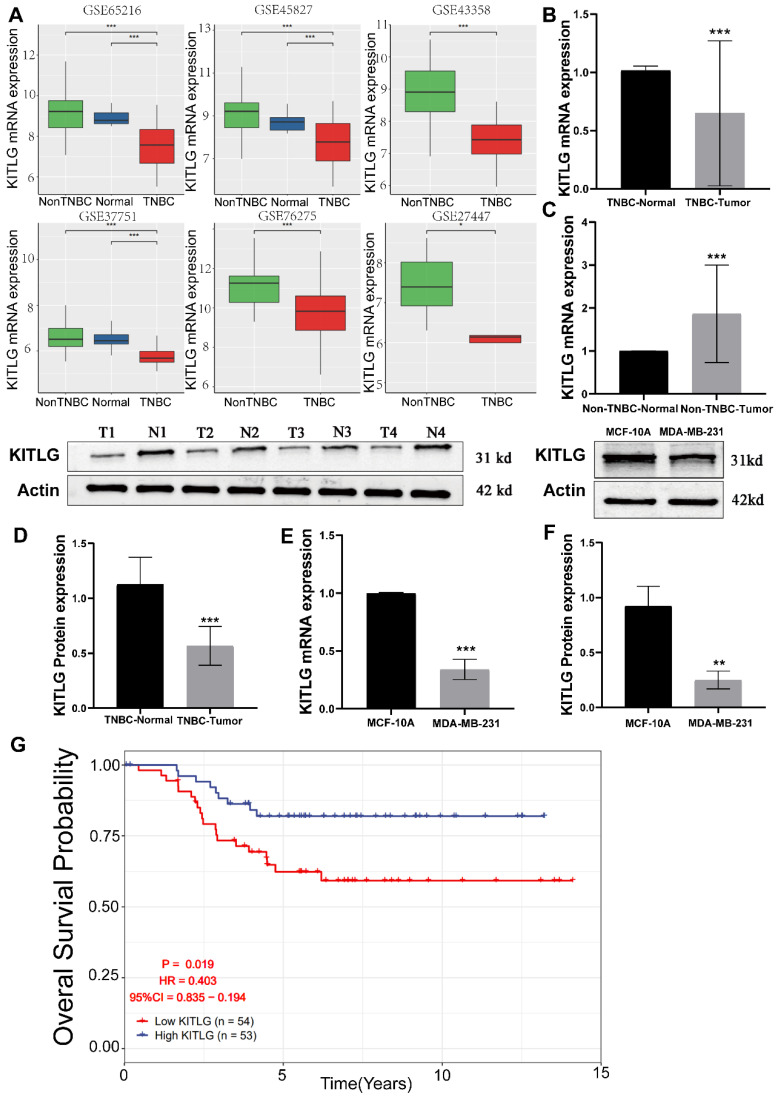
Low expression of KITLG correlates with TNBC tumorigenesis. (A)KITLG analysis of variance in multiple GEO datasets (non-TNBC: non-triple negative breast cancer, TNBC: triple-negative breast cancer, *p< 0. 05, **p < 0. 01, ***p < 0. 001); (B) Expression level of KITLG mRNA in clinical TNBC samples (***p<0. 001 vs normal); (C) Expression level of KITLG mRNA in clinical non-TNBC samples (***p<0. 001 vs normal); (D) KITLG protein expression levels in clinical TNBC specimens (***p<0. 001 vs Normal); (E) KITLG mRNA expression in triple-negative breast cancer cells and normal breast epithelial cells (***p<0. 001 vs MCF-10A); (F) KITLG protein expression in triple-negative breast cancer cells and normal breast epithelial cells (**p<0. 01 vs MCF-10A); (G). Survival analysis of KITLG in triple-negative breast cancer in GEO database. Kaplan-Meier survival curves of overall survival in the GSE103091 cohort.

The reduction of KITLG expression is associated with the onset and advancement of TNBCs, and specifically, it is connected to an unfavorable prognosis. By exerting its effects through autocrine or paracrine signaling, it plays a pivotal role in facilitating aggressive tumorigenic processes, thus critically influencing to the augmentation of cancerous cell proliferation, movement, and infiltration. 11-13. Prior research has extensively documented the close association between KITLG and the progression of various types of cancers 14, 15. However, there are yet to be any studies that directly connect the functionality of KITLG with the progression or metastasis of TNBCs. Intrigued by the potential metastatic functions of KITLG, we brought this gene into focus for our follow-up work delineated below. To delineate the influence of KITLG on prognosis, we noted that its expression was markedly diminished in triple-negative breast cancers, founded on multifarious independent analyses of GEO data (Figure 1A). Pivoting to the clinical importance of KITLG expression, we analyzed TNBC and non-TNBC samples collected from the First Affiliated Hospital of Guangxi Medical University. Remarkably, we found decreased expression of KITLG mRNA levels in more than half of the TNBC samples and increased expression in most of the non-TNBC samples (Figure 1B, C). Further, protein levels of KITLG in TNBC tissues were considerably reduced compared to paired adjacent normal breast tissues (Figure 1D). Based on the differential expression of KITLG between TNBC and Non-TNBC, we hypothesized that KITLG expression might have implications for breast cancer malignancy, progression, and prognosis. Results derived from the application of WB and PCR methods to detect KITLG expression in triple-negative breast cancer cells, specifically MDA-MB-231, echoed our earlier findings (Figure 1E, F). A probe into the prognostic importance of KITLG expression in TNBC patients revealed that the downregulation of KILTG was significantly linked to decreased OS in the GEO (Figure 1G). Moreover, our result declared that the poorerly differentiated TNBC shows lower KITLG expression (Table S2). Other clinical features showed no association with KITLG expression, including age, menopausal, and TNM stage. In sum, these revelations argue strongly for the contribution of KITLG to the clinically relevant characteristics of TNBC as well as its crucial role in TNBC metastasis.

### Overexpression of KITLG inhibits cell proliferation, invasion, and migration in TNBC

**Figure 2:**
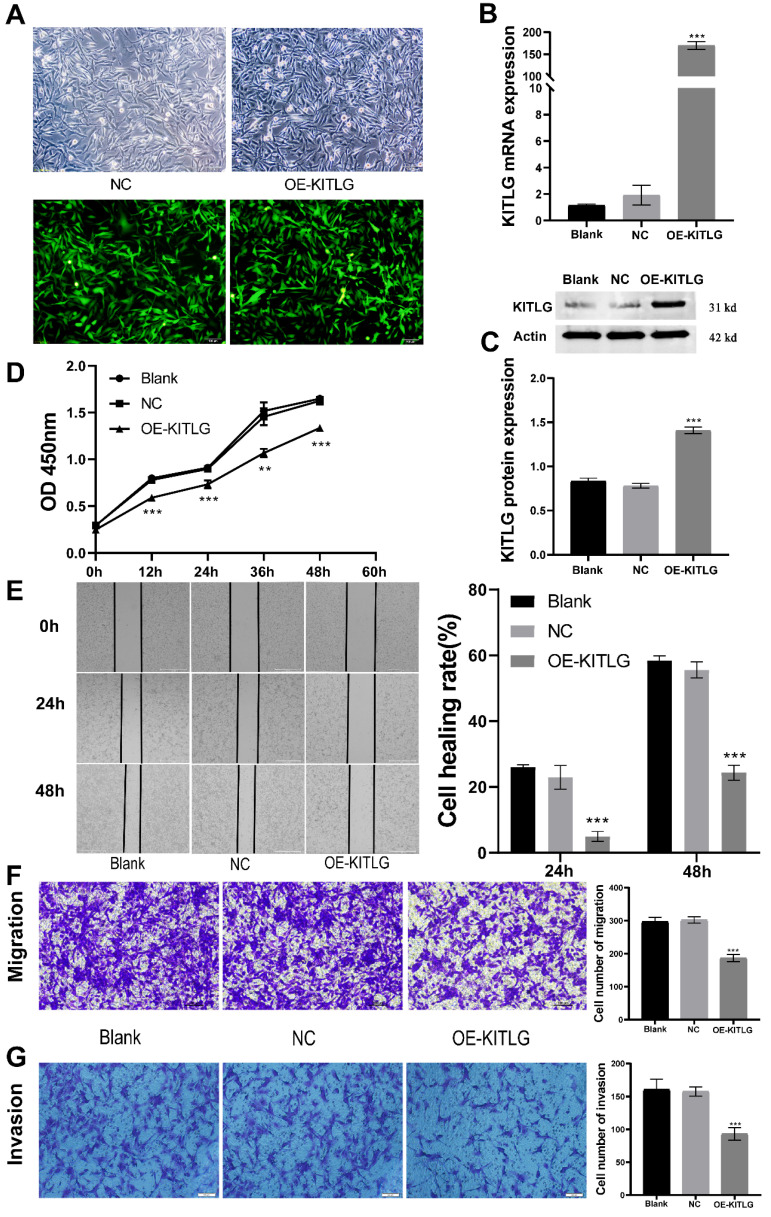
Overexpression of KITLG inhibits cell proliferation, invasion, and migration in TNBC. (A)Fluorescence expression of MDA-MB-231 cells 48h after transfection with lentivirus; (B) Expression levels of KITLG mRNA in cells of each experimental group after transfection (***P<0.001 when compared with Blank group or NC group, respectively); (C) KITLG protein expression in cells of each experimental group after transfection (***P<0.001 when compared with Blank group or NC group, respectively); (D) Line figure of OD value at each time point of each group after transfection (**P<0.01 ***P<0.001 when compared with Blank group or NC group, respectively); (E) Scratch areas of cells in each group after transfection at 24h and 48h; Cell healing rate at 24h and 48h after transfection (***P<0.001 when compared with Blank group or NC group, respectively); (F) Cells passing through the chamber and the number of cells in each group in Transwell migration experiment (***P<0.001 when compared with Blank group or NC group, respectively); (G) Cells passing through the chamber and the number of cells in each group in the Transwell invasion experiment (***P<0.001 when compared with Blank group or NC group, respectively).

Amplification of KITLG restrains cellular propagation, invasiveness, and migration within TNBC. To delineate KITLG's function in cancer progression, we stably enhanced the expression of KITLG in MDA-MB-231 cells via lentiviral infestation. As depicted in Figure 2A, by implementing real-time fluorescence quantitative PCR analysis, we observed the mRNA expression of KITLG in cells bolstered with enhanced KITLG (designated as OE) was notably superior to the KITLG levels in both the blank control (Blank) and negative control (NC) subsets. These findings were corroborated by both Western blotting and PCR (Figure 2B, C). Subsequently, we employed the CCK-8 assay to evaluate if the augmented expression of KITLG impacted MDA-MB-231 cells' propensity to proliferate. After successive measurements at intervals of 12, 24, 36, and 48 hours, we discerned that the OD metrics of the OE-KITLG subset were considerably diminished in comparison with both the Blank and NC groups. The CCK-8 assay implied that the increased expression of KITLG provokes a decline in TNBC's proliferation rate (Figure 2D).

Moreover, to gauge the repercussions of KITLG overexpression on the migratory and invasive capabilities of triple-negative breast cancer cells, we instituted cell-scratching and Transwell migration and invasion procedures. The results from both cell migration and invasion and Wound-healing assays (Figure 2E) disclosed that elevating the expression of KITLG within MDA-MB-231 cells distinctly curtailed cellular migration and invasion (Figure 2F, G). Combining these observations implies that the overexpression of KITLG significantly blunts the proliferative and migratory capability of TNBC. Our subsequent evaluation considered the influence of KITLG on cell migration and intrusion. Wound-healing assays conveyed that diminishing KITLG expression within MDA-MB-231 cells substantially suppresses cellular drift. Analogous patterns were observed in Transwell assays, demonstrating a negative correlation between KITLG expression and TNBC cells' migration and invasive attributes.

### KITLG promotes apoptosis in triple-negative breast cancer cell lines

**Figure 3:**
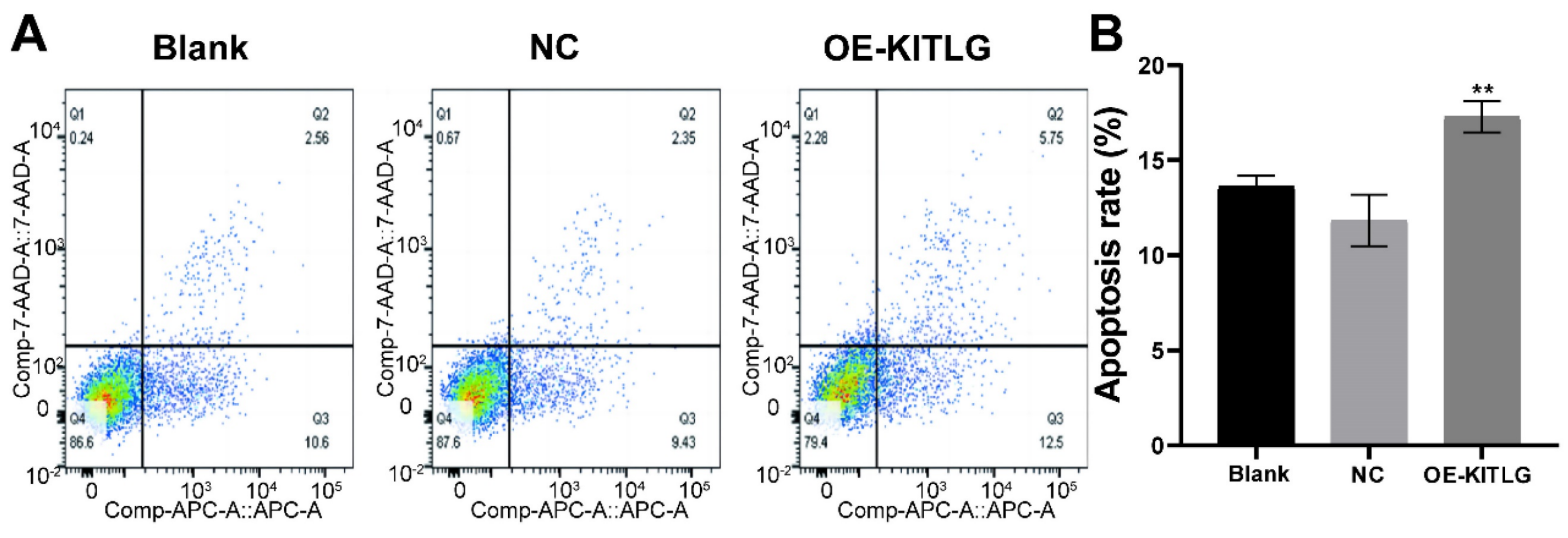
KITLG promotes apoptosis in triple-negative breast cancer cell lines. (A) Apoptosis of cells in each group in apoptosis experiment; (B) Histogram of apoptosis rate in Q2+Q3 of each group in cell apoptosis experiment (**P<0.01 when compared with Blank group or NC group, respectively).

Our extensive exploration of the impact of KITLG has shed new light on its intriguing role as a potential tumor suppressor, particularly through the activation of apoptotic pathways in triple-negative breast cancer cell lines. We further investigated the effect of KITLG on tumor suppressor function, for example in cell death assays. We also examined the effect of KITLG on the apoptosis of MDA-MB-231 cells. The results of Annexin V apoptosis detection showed that the overexpression of KITLG increased the apoptosis of MDA-MB-231 cells. We concentrated our efforts on MDA-MB-231 cells in this investigation, thoroughly examining the influence of KITLG on apoptosis within these cells (Figure 3A, B). Insights gleaned from Annexin V apoptosis experiments provide compelling evidence that an upsurge in KITLG expression precipitates an increase in cell apoptosis specifically within the MDA-MB-231 cell line.

These findings further underscore the indispensable role of KITLG as a promoter of apoptosis within triple-negative breast cancer cells and invite further examination of its potential therapeutic application in combating this prevalent disease.

### KITLG enhances the cancer-inhibiting effects of PI3K/AKT through modulating c-KIT activity and inhibits the progression of triple-negative breast cancer by promoting the apoptotic function of Casp3/9

**Figure 4:**
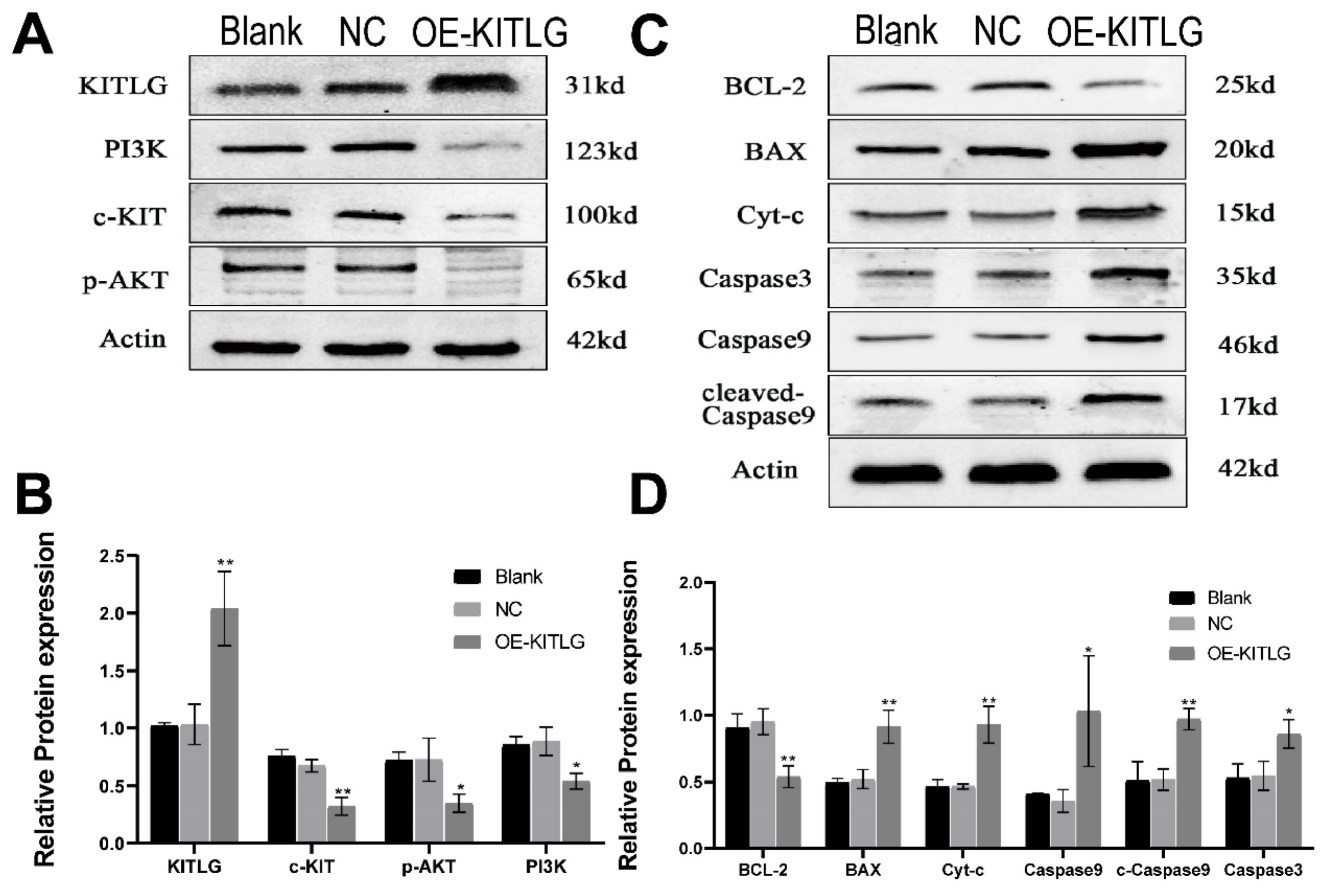
KITLG enhances the cancer-inhibiting effects of PI3K/AKT by regulating c-KIT activity and inhibits the progression of triple-negative breast cancer by promoting the apoptotic function of Casp3/9. (A) Expression of c-KIT /PI3K pathway after overexpression of KITLG; (B) Quantitative histogram of c-kit /PI3K pathway protein after overexpression of KITLG (*P<0.05 **P<0.01 when compared with Blank group or NC group, respectively); (C) expression of apoptosis-related indicators after overexpression of KITLG; (D) quantitative histogram of apoptosis-related indicators protein after overexpression of KITLG (*P<0.05 **P<0.01 when compared with Blank group or NC group, respectively).

The protein KITLG augments the anticancer effects of PI3K/AKT by modulating c-KIT activity and retards the growth of TNBC by enhancing the apoptotic activity of Casp3/9. Literature suggests KITLG exerts a regulative influence on KIT, also known as the human stem cell factor, which functions as a ligand for the c-KIT transmembrane tyrosine kinase receptor (KIT). However, studies highlighting the specific interaction between these two in the context of TNBC are scant. Recently in our laboratory, we achieved stable overexpression of KITLG in TNBC cell lines and observed via WB assays that overexpression significantly diminished KIT expression. Furthermore, WB assays authenticated the concomitant decrease in KIT protein expression. Collectively these findings suggest that KITLG may negatively regulate KIT-transcriptional activity in TNBC. c-KIT is a unique receptor for KITLG, and once they form a complex, downstream pathways such as PI3K/AKT are activated, which affect fundamental cellular functions including proliferation, migration and survival. Evidence from the KEGG database coupled with previous literature substantiates the involvement of KIT in PI3K/AKT-mediated signal transduction mechanisms. As a part of our investigation into the molecular machinery underpinning KITLG-induced apoptosis, we probed the regulation of downstream genes associated with this pathway by KITLG. Transfecting cells with KITLG caused a suppression in the mRNA levels of genes related to this pathway, such as PI3K, and AKT. Supporting this, consistent reduction at the protein level affirmed that PI3K and AKT levels drop after KITLG overexpression (Figure 4A, B). Flow cytometry results indicated a rise in apoptosis once KITLG was overexpressed, warranting further exploration into the role of KITLG in inhibiting TNBC cells. We posited that modulation of apoptosis-related genes could mechanize the regulatory effect of KITLG on apoptosis. Checking the protein levels of apoptosis-related genes BCL-2, BAX, Caspase3, Caspase9, and Cyt-C showed that KITLG overexpression notably increased the protein level of Caspase3/9 (Figure 4C, D).

Overall, the data suggest that KITLG may regulate TNBC progression, and instigate apoptosis, and its modus operandi may be strongly linked to the PI3K-AKT and Caspase-3/Caspase-9 pathway.

### KITLG inhibits the TNBCs tumor growth in vivo

In pursuit of comprehending the effects of KIT ligand (KITLG) on the development of TNBC in vivo, a xenograft experiment entailing subcutaneous tumor grafting was conducted. This experimental process utilized MDA-MB-231 cells, wherein overexpression of KITLG was deliberately induced in mice.

**Figure 5:**
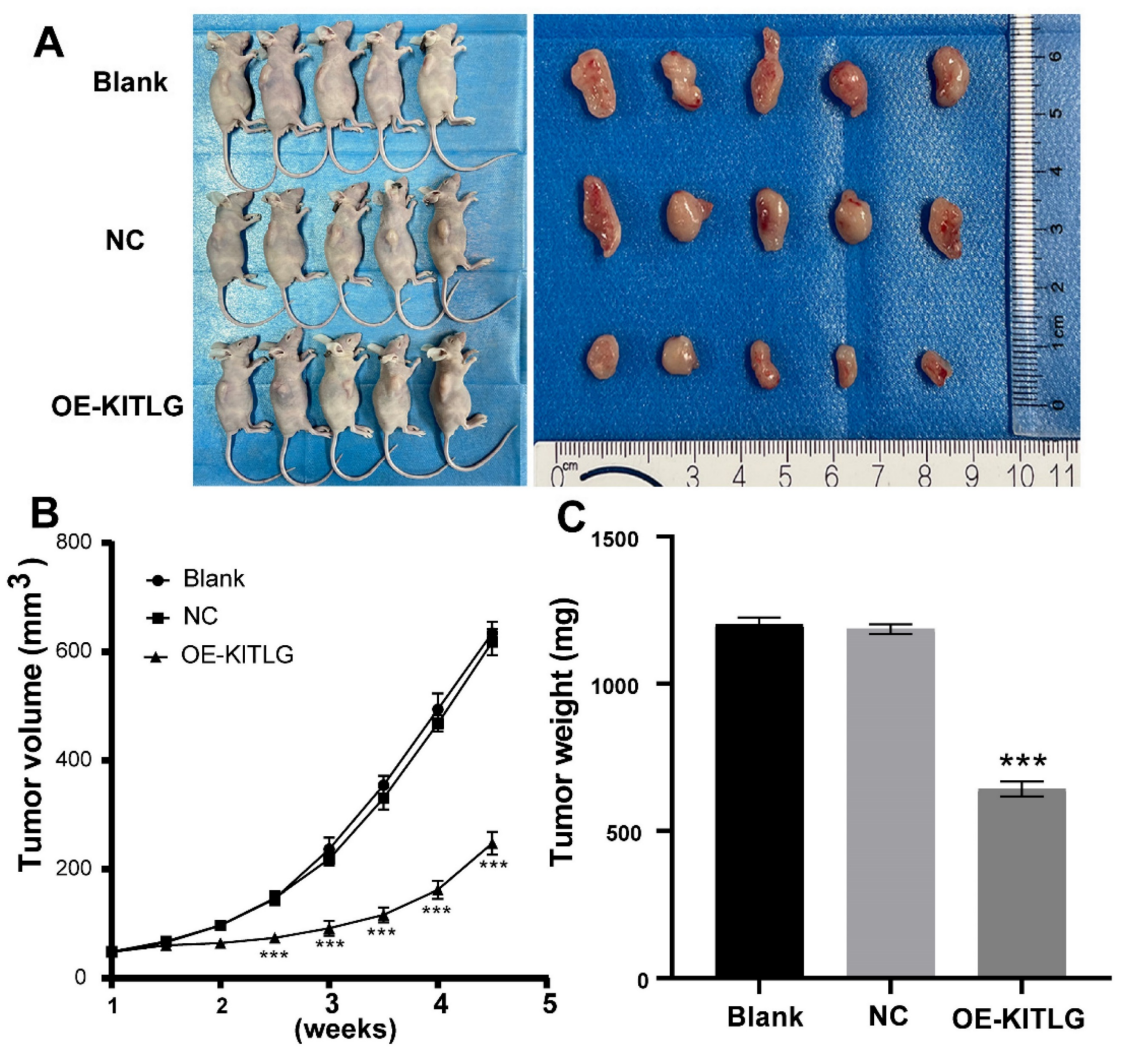
KITLG inhibits the TNBCs tumor growth in vivo. (A) Transplanted tumor models and tumor specimens of nude mice in each group after transfection; (B) Tumor volume growth curve of transplanted mice in each group after transfection (***P<0.05 when compared with blank group or NC group, respectively); (C) Tumor weight histogram of transfected nude mice in each group (***P<0.05 when compared with blank group or NC group, respectively).

To validate the effects, measurements relating to the body weight of the nude mice as well as the dimensions and weight of the induced tumors were noted post-establishment of a subcutaneous transplantation tumor model in the said mice. During the incubation period, the body weight of the mice, alongside the tumor volume, was periodically monitored, thereby enabling the construction of tumor growth volume curves. Following a period analogous to 28 days, the mice were euthanized, after which the tumors were excised and weighted to enable the calculation of the tumor suppression rate. As illustrated in Figures (Figure 5A, B, C), a significant decrease was noted in the volume and mass of tumors observed in the group draped as OE-KITLG, about the Blank and NC groups. Collectively, these findings denote a pivotal potential role of KITLG in perturbing the growth of TNBCs in vivo.

## Discussion

Breast cancer imposes a significant health burden on women, predominantly due to high morbidity and mortality rates. Even with adjunct chemotherapy, the five-year survival rate remains below 30% in patients with metastatic forms of the disease^16^. Breast cancer has become the predominant malignancy among women in China, with the highest rates of occurrence and ranking as the second leading cause of cancer-related fatalities within this population.

Thanks to advancements in medical imaging and diagnostic testing, the early detection of breast cancer has substantially improved, leading to a substantial increase in diagnosis rates. Despite this, treatment strategies for breast cancer to date primarily rely on multimodal approaches which include chemotherapy, endocrine therapy, targeted therapies, surgical interventions, and radiation therapy^17^. While significant advancements in treatment modalities have generally improved postoperative survival rates, recurrence and metastasis remain prevalent and are currently the leading cause of death in postoperative breast cancer patients. This absence generally limits the success of targeted therapies due to the inherently high heterogeneity of TNBC^18^. In the field of TNBC management, systemic treatments are predominantly chemotherapy driven. However, conventional postoperative adjuvant radiotherapy is relatively ineffective, contributing to a significant postoperative recurrence rate, peaking as high as 40%^19^. With conventional adjuvant radiotherapy following surgery consistently underperforming, the need for novel therapeutic strategies and biomarkers is increasingly urgent to enhance the prognosis and overall quality of life in patients diagnosed with TNBC. Diverse stromal cells within the hematopoietic microenvironment produce cytokines, which are novel proteins that play a crucial role in regulating the vital activities of hematopoietic stem cells through autocrine or paracrine mechanisms. Two types of these cytokines exist, categorized based on their ability and proficiency at promoting stem cell proliferation and differentiation. Proteins like SCF, FL, IL-1, and IL-12 stand out for their ability to enhance proliferation without compromising stemness. By activating the c-KIT tyrosine kinase receptor, the KIT proto-oncogene ligand (KITLG) induces various biological effects^4^. Specifically, in uveal melanoma, KITLG-dependent stimulation facilitates the proliferation and transformation of uveal melanoma cells via SCF/c-KIT autocrine loop activation^20^. Moreover, Elevated expression of KITLG has been associated with the pathological advancement of various malignancies, such as neurofibromatosis^21^, glioma, and papillary thyroid carcinoma 14. Furthermore, KITLG is now considered characteristic of WHO AB thymoma, potentially signifying a novel marker for differentiating WHO AB thymoma from other thymic tumors^22^. Recent investigations suggest that KITLG inhibits cancer through the induction and Amplification of multiple signaling pathways^23^-^25^. Despite the intricate nature of KITLG's mechanistic role in cancer, the aforementioned studies unequivocally designate KITLG as a key factor in cancer progression, thereby guiding our research orientation. Notably, the expression pattern, diagnostic, and prognostic relevance of KITLG in TNBC remains unexplored, underlining the potential significance of investigating KITLG's association with this specific cancer type. Excessive and unregulated multiplication of cells is a distinguishing characteristic of cancer cells, resulting in the development of a malignant phenotype that can significantly affect prognoses. Recent developments in cancer treatments have focused on inhibiting this cell proliferation. At the very core of this, we have KITLG, a pivotal regulator of cells that plays a key role in lifecycles, survival, and multiplication. When misregulated in cancer cells, this leads to abnormal proliferation. The primary reason for breast cancer patient fatality is metastasis, a multi-step process wherein tumor cells spread from the initial lesion to distant sites, creating secondary growths. Our research demonstrated that overexpressing KITLG reduces proliferation, migration, and invasion in MDA-MB-231 breast cancer cells. Experiments conducted with lab animals also confirmed that KITLG overexpression meaningfully curtailed the tumorigenic ability of the mice. Collectively, these results hint at KITLG's role in restraining cell proliferation, migration, and invasion in triple-negative breast cancer. The activation of the PI3K/AKT signaling pathway is a prevalent cause of human cancers and a pivotal pathway responsible for cancer development and progression^26^. This pathway can significantly influence cellular metabolism by direct regulation of nutrient transport proteins and metabolic enzymes, or by controlling transcription factors that regulate the expression of key metabolic pathway components. This, in turn, promotes the malignant behaviors of cancer cells. Numerous studies have underscored the regulatory role of the PI3K/AKT pathway in cancer.

Apoptosis is primarily controlled by the BCL-2 family, the Caspase family, and the oncogene P53, and is governed through mitochondrial and death receptor pathways^27^. In the mitochondrial apoptosis pathway, intracellular pro-apoptotic factors, such as BAX, alter mitochondrial membrane permeability. This change allows for pro-apoptotic factors like Cyt-c to enter the cytoplasm, where Cyt-c binds to Apaf-1 to create multimers. These complexes then bind to caspase precursors forming apoptotic bodies, which activate Caspase9 and subsequently downstream apoptosis-related proteins like caspase3, inducing apoptosis via the Caspase cascade reaction. In our study, the WB assay was employed to evaluate the relative protein expression of the c-KIT/PI3K signaling pathway and BCL-2/BAX apoptosis-related indices in cells of overexpressed KITLG (OE), negative control (NC), and blank control (Blank) groups. The purpose was to explore the roles of the KITLG gene in triple-negative breast cancer (TNBC). Empirical evidence increasingly points to the PI3K/AKT signaling pathway's regulatory role in cancer. Prior research in breast cancer indicates that PI3K/AKT pathway inhibition following lncRNA GHET1 knockdown reduces MCF-7 breast cancer cell growth and metastasis. Under the influence of KITLG, PI3K can activate via direct binding to Tyr-721 on c-KIT and indirect binding to the tyrosine-phosphorylated junction protein. AKT, a serine-threonine kinase functioning downstream of PI3K kinase, modulates the cell cycle and proliferation and suppresses pro-apoptotic signals.

In previous studies, KITLG has been responsible for elevated Bcl-2 and Bcl-XL protein levels in mast cells and cortical neurons, particularly in those reliant on Akt and ERK signaling. This upregulation then protects cells against apoptosis. The involvement of PI3K-AKT in the growth and development of various cancer cells is accepted, as is the irregular PI3K-AKT expression in breast cancer and the pivotal role of PI3K inhibitors in targeted breast cancer therapy. However, the specific role and regulation of PI3K-AKT in TNBC by KITLG remains unexplored.

Our research sought to unravel the impact of anomalous KITLG expression on the PI3K/AKT and Casp3/9 apoptotic pathways. We prove that KITLG increases programmed cell death in breast cancer cells and augments apoptosis by stimulating the transcription of c-KIT genes. Results show that KITLG overexpression in TNBC cells suppresses the protein expression of the c-KIT/PI3K pathway and apoptosis inducers, thereby inhibiting TNBC cell proliferation, migration, invasion, and apoptosis.

While our study has crucial limitations, such as the inadequate sample size which restricts confirmation of bioinformatics findings through clinical tissues, we are mindful of the necessity for future experiments to expand the sample size. Experiments conducted on nude mice have demonstrated the capacity of KITLG to impede the progression of TNBC. However, further investigations are imperative to ascertain its impact on the metastasis of TNBC in vivo. Our forthcoming research will explore the molecular mechanics of KITLG within triple-negative breast cancer by strategically integrating bioinformatics predictions with fundamental experiments. Conclusively, we recognize the valuable contribution of this study in highlighting KITLG as a significant biomarker or a prospective target in the management of TNBC. This specific field still lacks targeted therapeutic approaches, and this research fills that gap.

## Supplementary Material

Supplementary tables.

## Figures and Tables

**Table 1:** Primer sequences.

Gene Name	Primer sequences (5'-3')
KITLG	Forward primer ATTGTCAGACAGCTTGACTGATC
	Reverse primer GGTTCTGGGCTCTTGAATGATTT
GAPDH	Forward primer AATCAAGTGGGGCGATGCTG
	Reverse primer GCAAATGAGCCCCAGCCTTC
