# Supplementary material for: Exploring the biological behavior and underlying mechanism of KITLG in triple-negative breast cancer
Source: J Cancer. 2024 Jan 1;15(3):764–75. doi: 10.7150/jca.90051 (PMC10777047; doi:10.7150/jca.90051)
Supplement: Supplementary file 1 — Supplementary tables. [file jcav15p0764s1.pdf]

Supplementary materials

Table S1 The detail sample information of six TNBC cohort

GEO ID	TNBC	no-TNBC	Normal
GSE65216	55	98	11
GSE45827	41	89	11
GSE37751	14	43	47
GSE43558	17	40	--
GSE27447	5	14	--
GSE76275	198	67	--
GSE103091	107	--	--

Table S2 The relationship between clinical features and KITLG expression level in TNBC

Clinical Feature	Level	KITLG	P Value
Age	>55	9.77 ± 1.2	0.8
	<=55	9.73 ± 1.33	
Menopausal status	Pre-menopausal	9.76 ± 1.32	0.985
	Menopausal	9.75 ± 1.3	
	Post-menopausal	9.79 ± 1.22	
Differentiated status	Poor	9.45 ± 1.23	0.005
	Moderate	10.08 ± 1.31	
	Well	10.5 ± 0.89	
T stage	T1	9.65 ± 1.15	0.775
	T2	9.79 ± 1.29	
	T3	9.65 ± 1.3	
	T4	10.11 ± 1.06	
N stage	N0	9.65 ± 1.2	0.192
	N1	10.01 ± 1.29	
	N2	9.6 ± 1.2	
	N3	10.37 ± 1.35	
	NX	9.58 ± 1.3	
M stage	M0	9.76 ± 1.26	0.859
	M1	10.2 ± 2.21	
	MX	9.71 ± 1.26	
